# Supplementary material for: Genomic Characterization of Multidrug-Resistant Pathogenic Enteric Bacteria from Healthy Children in Osun State, Nigeria
Source: Microorganisms. 2024 Mar 1;12(3):505. doi: 10.3390/microorganisms12030505 (PMC10974654; doi:10.3390/microorganisms12030505)
Supplement: Supplementary file 1 [file microorganisms-12-00505-s001.zip › JessicaUwanibe_Supplementary T1_Microorganisms MDPI.pdf]

Table S1: Whole Genome Sequencing Parameters of Sequenced Isolates.

Sample ID	Bracken_taxon	Bracken _taxo_ratio	est_genome_ ratio	Est_sequenncing _depth	Est_genome_le nght	BUSCO_summary	N50
OL31 NF	Enterobacter hormaechei	82.47	1.15	126.48	5.8MB	C:96.8%[S:96.8% ,D:0.0%],F:1.6%, M:1.6%,n:124	20359
OL19 NF	Citrobacter FDAARGOS_156	21.05	-	-	4.5MB	C:91.9%[S:91.9% ,D:0.0%],F:6.5%, M:1.6%,n:124	12708
OE73 NF	Klebsiella pneumonia	93	1.16	157	6.6MB	C:98.3%[S:93.5% ,D:4.8%],F:0.0%, M:1.7%,n:124	225101
OE71 NF	Klebsiella quasivariicola	71.7	0.98	136	5.6MB	C:98.4%[S:98.4% ,D:0.0%],F:0.0%, M:1.6%,n:124	55207
OE54 NF	Klebsiella pneumonia	98.07	0.98	37.18	5.5MB	C:75.0%[S:75.0% ,D:0.0%],F:18.5% ,M:6.5%,n:124	6186
OE36 NF	Escherichia coli	99.49	1.04	44.47	5.3MB	C:100.0%[S:100. 0%,D:0.0%],F:0.0 %,M:0.0%,n:124	101434
OE28 NF	Escherichia coli	98	1.08	86.28	6MB	C:77.4%[S:74.2% ,D:3.2%],F:17.7% ,M:4.9%,n:124	3035
J21	Escherichia coli	98.66	0.99	866.37	5.1MB	C:100.0%[S:96.8%, D:3.2%],F:0.0%,M:0 .0%,n:124	84931
OL13 NFPC 1	Escherichia coli	94	1.19	93.89	6.6MB	C:100.0%[S:79.0 %,D:21.0%],F:0.0 %,M:0.0%,n:124	57338
OL44 NF	Escherichia coli	96	1.3	80.1	7.2MB	C:71.0%[S:71.0% ,D:0.0%],F:24.2% ,M:4.8%,n:124	2615
OE75 NF	Klebsiella pneumnia	95.8	1.52	62.44	8.5MB	C:91.9%[S:88.7% ,D:3.2%],F:5.6%, M:2.5%,n:124	8617
OE43 NF	Enterobacter cloacae	84.19	3.03	142.46	15.4MB	C:99.2%[S:37.9% ,D:61.3%],F:0.0% ,M:0.8%,n:124	2192
OE41 NF	Escherichia coli	73.87	2.15	115.42	11MB	C:96.0%[S:85.5% ,D:10.5%],F:4.0% ,M:0.0%,n:124	2419

C- Complete BUSCOs; **S**- Complete and single-copy BUSCOs; **D**- Complete and duplicated BUSCOs; **F**- Fragmented BUSCOs; **M**- Missing BUSCOs; **n**- Total BUSCO group searched.
